# Surgical Outcomes and Differences in Values of Ocular Parameters Following Vitrectomy for Macular Hole over a 10-Year Period

**DOI:** 10.3390/jcm15020570

**Published:** 2026-01-10

**Authors:** Tomohiko Torikai, Hiromi Ohara, Jun Takeuchi, Tadashi Yokoi, Yuji Itoh, Takashi Koto, Atsushi Shiraishi, Makoto Inoue

**Affiliations:** 1 Kyorin Eye Center, Kyorin University School of Medicine, 6-20-2 Shinkawa, Mitaka 186-8611, Japan; tomohiko-torikai@ks.kyorin-u.ac.jp (T.T.); hiromi-ohara@ks.kyorin-u.ac.jp (H.O.); jun.takeuchi622@outlook.com (J.T.); tadashi-yokoi@ks.kyorin-u.ac.jp (T.Y.); koto@ks.kyorin-u.ac.jp (T.K.); 2 Department of Ophthalmology, Graduate School of Medicine, Ehime University, Toon 791-0295, Japan; shiraia@m.ehime-u.ac.jp

**Keywords:** macular hole, surgical outcomes, axial length, inverted internal limiting membrane, vitrectomy

## Abstract

**Background/Objectives:** We aimed to compare the surgical outcomes of macular hole (MH) cases and evaluate how the axial length (AL) affected the outcomes. **Methods**: Six hundred and sixty-three eyes with MHs that underwent vitrectomy over the past 10 years were reviewed. The changes in AL were compared to those of 1948 eyes with idiopathic epiretinal membranes (ERMs) operated on during the same period. The MH cases for the 5 years from 2014 to 2018 were designated as the MH2014 group, and those from 2019 to 2023 as the MH2019 group. The ERM cases were divided similarly into the ERM2014 and ERM2019 groups. The clinical characteristics of the cases and surgical outcomes were compared. **Results**: The MH diameter, closure rate, and baseline and postoperative visual acuity were not significantly different. The use of the inverted internal limiting membrane flap technique was significantly higher in the MH2019 group (58%) than in the MH2014 group (24%, *p* < 0.001). The mean AL was significantly longer in the MH2019 group (25.2 ± 2.4 mm) than in the MH2014 group (24.6 ± 2.1 mm, *p* = 0.004). The incidence of myopic MHs with AL ≥ 26 mm and AL ≥ 30 mm was higher in the MH2019 group (30.9%, *p* = 0.008, 6.4%, *p* = 0.017, respectively). There was a significant trend for longer ALs over 10 years in the MH group (*p* = 0.002), but not in the ERM group. **Conclusions**: The increased AL and the rising proportion of eyes with myopic MHs indicate that the patient profile of eyes with MHs has changed over the past decade.

## 1. Introduction

Vitreous traction on the retina caused by a contraction of the perifoveal vitreous cortex has been reported to be the cause of the development of idiopathic macular holes (MHs) [1,2]. In 1991, Kelly and Wendel [3] were the first to report that pars plana vitrectomy with a gas tamponade can successfully close MHs. The procedures used for these surgeries have greatly improved; for example, better surgical instruments and procedures, the use of internal limiting membrane (ILM) peeling, and the inverted ILM flap technique have all resulted in a greatly improved closure rate [4,5,6,7,8,9,10,11,12,13,14,15,16,17,18,19,20,21,22,23,24].

Johnson and Gass reviewed 158 eyes with developing or completed idiopathic MH in 1988. They reported that the average refractive error was +0.4 diopters (D) in the right eye and +0.5 D in the left eye [25]. Kobayashi and associates [26] evaluated 94 eyes of 91 patients with stage 3 or 4 MH and reported that the MHs developed at a significantly younger age in eyes with severe myopia. They reported that the average refractive error was emmetropia or hyperopia (axial length (AL) < 23.0 mm) in 60%, mild to moderate myopia (AL ≥ 23.0 mm but <26.0 mm) in 19%, and high myopia (AL ≥ 26.0 mm) in 21%. These findings indicated that MHs developed mostly in emmetropic or hyperopic eyes. Singh and associates [27] compared 40 patients with stage 3 or 4 MH to an age-matched control group in 2012. They reported that the mean AL was 23.62 mm in the MH patients, which was longer than the 23.09 mm in the control group. They suggested that the greater tension on the macula by the elongation of the AL may have played a role in the formation of the MHs [27].

In the Hisayama cohort study, the ALs of eyes examined in 2017 were significantly longer than those examined in 2005. The percentage of patients with an AL ≥ 26.5 mm increased from 3.6% to 6.0% during this period, indicating that the prevalence of myopia had increased in the Japanese population during those 13 years [28]. We considered the possibility that the increase in the myopic population may have affected the prevalence of myopic MHs in the Japanese population.

The inverted ILM flap technique has been used in eyes with large MHs > 400 μm and also in eyes with myopic MH because it is difficult to close MHs using conventional ILM peeling in highly myopic eyes [10,11,29]. It is possible that the number of cases in which the inverted ILM flap technique was used may have increased with the increase in myopia. However, to the best of our knowledge, there has not been a study on the changes in the AL and the prevalence of MHs.

Thus, the purpose of this study was to determine the AL and rate of increase in the prevalence of myopia in eyes with MHs during a 10-year period. To accomplish this, we examined the medical records of patients who had undergone vitrectomy over 10 years and collected data on the patient backgrounds and the surgical outcomes from their medical records.

## 2. Materials and Methods

A power analysis was conducted in G*Power (ver. 3.1.9.7) to detect the mean difference between two independent groups [30]. With a medium effect size (Cohen’s d = 0.5), an alpha level of 0.05, and a target power of 0.95 (95%), the analysis indicated that 110 participants per group were required. Accordingly, the study was designed to include a total of 220 participants, meeting the required sample size.

The procedures used in this retrospective study in a single academic institution were approved by the Institutional Review Committee of the Kyorin University School of Medicine (R06-188). The procedures conformed to the tenets of the Declaration of Helsinki, and all of the patients had signed an informed consent form prior to the surgery. They had all consented to our review of their medical records and to the anonymized use of their data in medical publications.

### 2.1. Subjects

We studied 663 eyes of 640 Japanese patients who had undergone vitrectomy for an MH at the Kyorin University Hospital from April 2014 to March 2024. All eyes were followed for at least 3 months after the surgery. Only patients who underwent initial vitrectomy were included, and those who underwent multiple vitrectomies and those who had an MH retinal detachment were excluded. The patients who had undergone vitrectomy for MH in a 5-year period from April 2014 to March 2019 were placed in the MH2014 group, and those from April 2019 to March 2024 were placed in the MH2019 group. One year was defined as the period between April of one year to March of the following year.

The idiopathic MHs were classified according to Gass’s classification [2]. For the comparisons of the changes in the AL, 1948 eyes that were diagnosed with an epiretinal membrane (ERM) and had undergone vitrectomy during the same period were placed in the control group. The 998 eyes that underwent vitrectomy for an ERM in a 5-year period from April 2014 to March 2019 were placed in the ERM2014 group and the 950 eyes from April 2019 to March 2024 were placed in the ERM2019 group.

### 2.2. Surgery

Surgery was performed by experienced vitreoretinal surgeons using a 25-gauge or a 27-gauge vitrectomy system (Constellation Vision System, Alcon Laboratories, Fort Worth, TX, USA, or EVA, D.O.R.C. International, Zuidland, The Netherlands). A cataractous lens was removed by phacoemulsification with an implantation of an intraocular lens in patients > 50 years of age. Core vitrectomy was performed, and then a posterior vitreous detachment was created if it was not present. Triamcinolone acetonide (MaQaid^®^, Wakamoto Pharmaceutical Co., Ltd., Tokyo, Japan or Kenacort-A^®^, Bristol Pharmaceuticals KK, Tokyo, Japan) was injected intravitreally to make the vitreous gel more visible. Brilliant Blue G (BBG) was used to make the ILM more visible. The ILM was peeled to an approximately 1.5-to-2-disc diameter around the MH and then either removed or inverted and placed over the open MH. The procedure was used was chosen by each surgeon according to the diameter of MH and the degree of myopia.

### 2.3. Evaluations of Clinical Data

The demographics of the patients were collected from the medical records. The number of patients, sex, laterality, age, presence or absence of a cataractous lens, and duration of the postoperative observation period were compared between the two groups. The decimal BCVAs were converted to logarithm of the minimum angle of resolution (logMAR) units for the statistical analyses.

We analyzed the best-corrected visual acuity (BCVA) in logMAR units, the minimum and base diameter of the MH, stage of the MH, and whether the inverted ILM flap technique was used. The minimum linear diameter and base diameter were adjusted with the AL using the linear scaling method: Corrected size = Measured size × (AL patient/AL_0_), where the AL of a patient is the individual AL and AL_0_ is the device-assumed AL (set to 24.0 mm for our analyses) [31]. The classification of MH was made according to the criteria set by the CLOSE study group [32]. The preoperative BCVA, that at 3 and 6 months, and that at the final visit were used for the analyses. The percentage of eyes with an initial and final closure of the MH, the AL, and the percentage of myopic MHs in the two groups were compared. The AL of eyes at each stage of the MH and the distribution of the changes in the AL between the MH2014 and MH2019 groups and between the ERM2014 and ERM2019 groups were compared. We also examined the incidence of myopic MHs (AL ≥ 26 mm or ≥30 mm) and evaluated the trend of the changes by the year.

Optical coherence tomography (OCT) was used to determine the status of the MH, and two models of spectral-domain OCT devices were used: the Spectralis^®^ OCT (Heidelberg Engineering, Heidelberg, Germany) and the swept-source OCT (DRI OCT-1 Atlantis^®^, TOPCON Corporation, Tokyo, Japan). The AL was measured by an optical biometer (OA-2000, TOMEY Corporation, Nagoya, Japan).

### 2.4. Statistical Analyses

All statistical analyses were performed using SPSS software (ver. 29.0.2.0; IBM Corp., Armonk, NY, USA) and R software (ver. 4.4.2; R Foundation for Statistical Computing, Vienna, Austria). *p* < 0.05 was taken to be significant, and the Shapiro–Wilk test was used to test the normality of the data distribution. Comparisons between groups were made using Mann–Whitney *U* tests and chi-square tests. In the chi-square test, standardized residuals with an absolute value of 2 or greater were considered statistically significant differences. Wilcoxon’s signed rank tests were used for the comparisons of the BCVA in logMAR units before and after the surgery. For comparisons of the ALs between the two groups, the Kruskal–Wallis test was used to determine whether the differences in the distribution were significant. Multiple comparisons were conducted using the Dunn tests with the R software when significant differences were found. In addition, the Jonckheere–Terpstra test in the R software was used to evaluate the year-to-year changes in the AL.

## 3. Results

### 3.1. Demographics of Participants

There were 349 eyes in the MH2014 group and 314 eyes in the MH2019 group, and 998 eyes in the ERM2014 group and 950 eyes in the ERM2019 group (Table 1). This study included cases that underwent vitrectomy during the study period, so each group contained cases involving both eyes (4.8% in MH2014 and 2.3% in MH2019, 5.8% in ERM2014 and 5.7% in ERM2019). The number of subjects in the MH2014 group, MH2019 group, ERM2014 group, and ERM2019 group (Wilcoxon–Mann–Whitney test) all exceeded the requirement of 110 subjects, as determined by the power analysis [30]. For the intergroup comparison of the proportion (chi-square test, effect size w = 0.3, degrees of freedom df = 5), the required sample size was also 220 cases. The sample sizes in both the MH and ERM groups in this study provided sufficient statistical power (Power > 0.95). These results suggest that the sample size in this study falls within a statistically valid range.

There were 128 men and 205 women in the MH2014 group and 122 men and 185 women in the MH2019 group. The difference in the sex distribution was not significant. The number of phakic eyes was 315 (90%) in the MH2014 group, which was significantly higher than that in the MH2019 group at 84% (*p* = 0.023). The follow-up period was 22.0 ± 23.6 months in the MH2014 group, which was significantly longer than that in the MH2019 group (9.5 ± 9.7 months, *p* < 0.001).

In the control ERM groups, there were 430 men and 513 women in the ERM2014 group, and 381 men and 518 women in the ERM2019 group (Table 1). The number of phakic eyes was 897 (90%) in the ERM2014 group and 825 (87%) in the ERM2019 group. The number of phakic eyes in the ERM2014 group was significantly greater than that in the ERM2019 group (*p* = 0.043). There were no significant differences in the age, laterality of the eyes, or sex distribution between the MH and ERM groups.

### 3.2. Diameter and Stage of Macular Hole (MH)

The average minimum MH diameter was 320 ± 175 μm in the MH2014 group and 297 ± 156 μm in the MH2019 group, and this difference was not significant (Table 2). The minimum diameter adjusted to the AL was 328 ± 186 μm in MH2014 and 309 ± 159 μm in MH2019. This difference was also not significant. The base diameter of the MH was 710 ± 336 μm in the MH2014 group and 687 ± 333 μm in the MH2019 group (*p* > 0.05). The base diameter adjusted with the AL was 731 ± 357 μm in the MH2014 and 717 ± 346 μm in the MH2019, neither of which were significant.

In the MH2014 group, the MH was at stage 1 in 21 eyes (6%), stage 2 in 53 eyes (15%), stage 3 in 158 eyes (45%), and stage 4 in 117 eyes (34%, Table 2). In the MH2019 group, there were 19 (6%) eyes at stage 1, 68 (22%) eyes at stage 2, 96 (31%) eyes at stage 3, and 131 (42%) eyes at stage 4. The difference in the number of cases at each stage in the two groups was significant (*p* = 0.001). Analysis of the adjusted standardized residuals showed that the number was significantly higher in the stage 3 eyes in the MH2014 group and in the stage 2 and 4 eyes in the MH2019 group.

### 3.3. Use of Inverted ILM Flap Technique and Macular Hole Closure Rates

In 2010, Michalewska et al. were the first to propose using the inverted ILM flap technique [11]. In the MH2019 group, 181 eyes (58%) were treated with the inverted ILM flap technique which was significantly higher than the 84 eyes (24%) in the MH2014 group (*p* < 0.001, chi-squared test). For eyes with an AL ≥ 26 mm, 72 of 97 eyes (74%) in the MH2019 group and 32 of 76 eyes (42%) in the MH2014 group underwent the inverted ILM flap technique. The use of the inverted ILM flap technique in eyes with an AL ≥ 26 mm was significantly higher in the MH2019 group (*p* < 0.001).

The initial MH closure rate was 97.7% in the MH2014 group and 99.0% in the MH2019 group, and the final MH closure rate was 98.6% in the MH2014 group and 100% in the MH2019 group. The difference in the closure rates between the two groups was not significant.

The initial MH closure rate in eyes with an AL ≥ 26 mm but less than 30 mm was 100% (77/77 eyes) in the MH2019 group and 95.5% (64/67 eyes) in the MH2014 group (*p* = 0.093, Fisher’s exact test). The final MH closure rate was 98.5% (66/67 eyes) in the MH2014 group. The inverted ILM flap technique was not used in the three eyes without an initial MH closure in the MH2014 group. The ILM was peeled off in two eyes, and the inverted ILM flap was partially detached without complete coverage of the MH in one eye. One eye in the MH2014 group achieved MH closure after a second surgery.

The initial MH closure rate in eyes with an AL ≥ 30 mm was 95.0% (19/20 eyes) in the MH2019 group and 77.7% (7/9 eyes) in the MH2014 group (*p* = 0.220, Fisher’s exact test). In the three eyes without an initial MH closure, the inverted ILM flap technique was not performed. One eye in the MH2019 group achieved an MH closure after a second surgery.

### 3.4. Visual Acuity Outcomes

**Figure 1:**
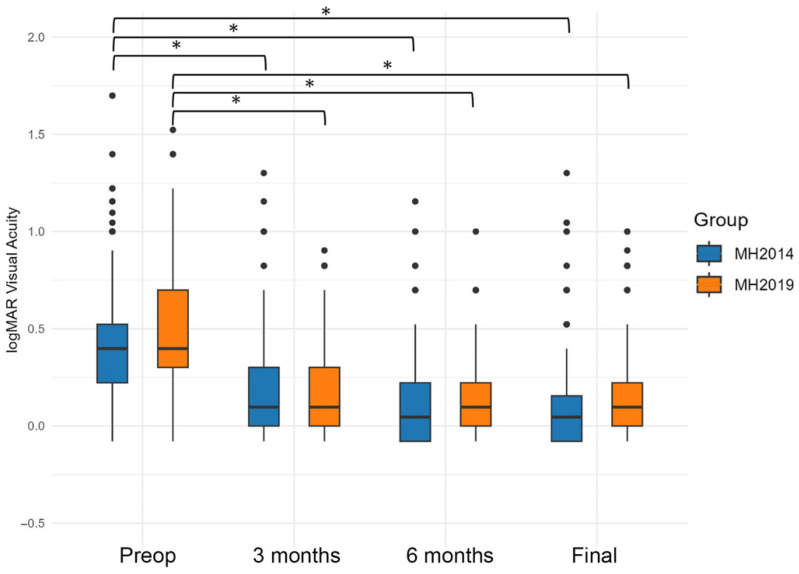
Visual outcome following vitrectomy on eyes with a macular hole (MH) in the MH2014 and MH2019 groups. The postoperative vision improved significantly in both groups. *; *p* < 0.05, Wilcoxon’s signed rank test.

The changes in the BCVA in the MH and ERM groups before surgery, at 3 and 6 months postoperatively, and at the last visit are shown in Figure 1. None of the eyes experienced severe vision loss caused by postoperative complications of endophthalmitis and retinal detachment. The preoperative BCVA was 0.45 ± 0.29 logMAR units in the MH2014 group (*n* = 349) and 0.46 ± 0.29 logMAR units in the MH2019 group (*n* = 314). The difference between the two groups was not significant (Table 2). The postoperative BCVA at 3 months was 0.15 ± 0.23 in the MH2014 group and 0.16 ± 0.21 in the MH2019 group, and both BCVAs were significantly better than that at the baseline (*p* < 0.001, *p* < 0.001, Wilcoxon signed-rank test, Figure 1). However, the difference between the two groups was not significant. The postoperative BCVA at 6 months was 0.12 ± 0.22 logMAR units (*n* = 272) in the MH2014 group and 0.13 ± 0.21 logMAR units (*n* = 180) in the MH2019 group. Both were significantly better than that at the baseline (*p* < 0.001, *p* < 0.001, Wilcoxon signed-rank test), but the BCVAs were not significantly different between the two groups. The BCVA at the last visit was 0.10 ± 0.22 logMAR units in the MH2014 group and 0.13 ± 0.22 logMAR units in the MH2019 group. The BCVA was significantly better in the MH2014 group (*p* = 0.008, Mann–Whitney *U* test), possibly due to the longer observation period in the MH2014 group (*p* < 0.001, Mann–Whitney *U* test, Table 1).

### 3.5. Axial Length (AL) and Macular Hole (MH) Stage

The mean AL in the MH2019 group was 25.2 ± 2.4 mm, which was significantly longer than that in the MH2014 group at 24.6 ± 2.1 mm (*p* = 0.004, Mann–Whitney *U* test, Table 3). The percentage of eyes with AL ≥ 26 mm in the MH2019 group was 30.9% (97 eyes), which was significantly higher than that in the MH2014 group at 21.8% (76 eyes, *p* = 0.008). The percentage of eyes with an AL ≥ 30 mm in the MH2019 group was 6.4% (20 eyes), which was significantly higher than that in the MH2014 group at 2.6% (9 eyes, *p* = 0.017).

The mean AL in the ERM2019 group was 24.6 ± 1.7 mm, which was significantly longer than the 24.5 ± 1.8 mm in the ERM2014 group (*p* = 0.028, Table 3). The percentage of eyes with an AL < 23 mm in the ERM2019 group was 15.8% (150 eyes), which was significantly lower than that in the ERM2014 group at 21.4% (*p* = 0.028, Table 4). However, the number of eyes with an AL ≥ 26 mm or ≥ 30 mm was not significantly different between the two groups (Table 3 and Table 4).

**Figure 2:**
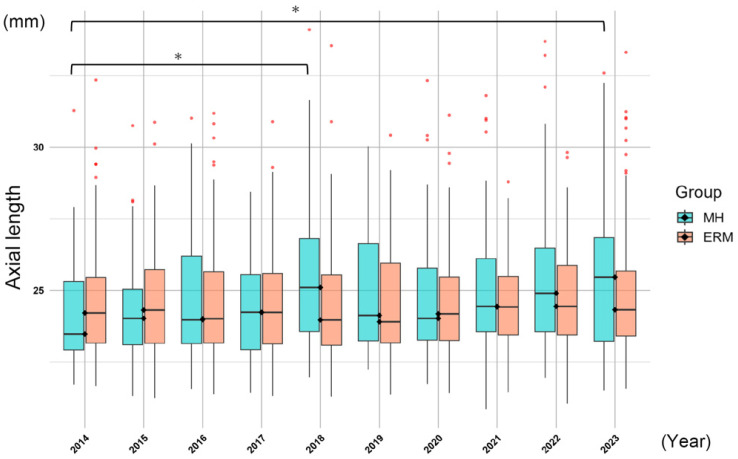
Annual changes in the axial length. In the MH group, there was a significant difference in the distribution of the AL over a 10-year period (*p* = 0.002, Kruskal–Wallis test). The AL at 2018 and 2023 in the MH group was significantly longer than that of the baseline at 2014 (*; *p* = 0.012, Dunn test with Holm correction). No significant difference in the distribution was observed in the ERM group (*p* = 0.343).

The mean ALs in the MH2019 group and the ERM2019 group were significantly longer than those in the MH2014 and ERM2014 groups. The results of the comparisons of AL by the year in the MH and ERM groups are shown in Figure 2. In the MH group, there was a significant difference in the distribution of ALs over the 10-year period (*p* = 0.002, Kruskal–Wallis test). In addition, in the MH group, there was a gradual increase in the AL from year to year (*p* < 0.001, Jonckheere–Terpstra test). No significant difference was found in the distribution in the ALs in the ERM groups (*p* = 0.343, Kruskal–Wallis test), but a similar gradual increase was observed (*p* = 0.017, Jonckheere–Terpstra test).

**Figure 3:**
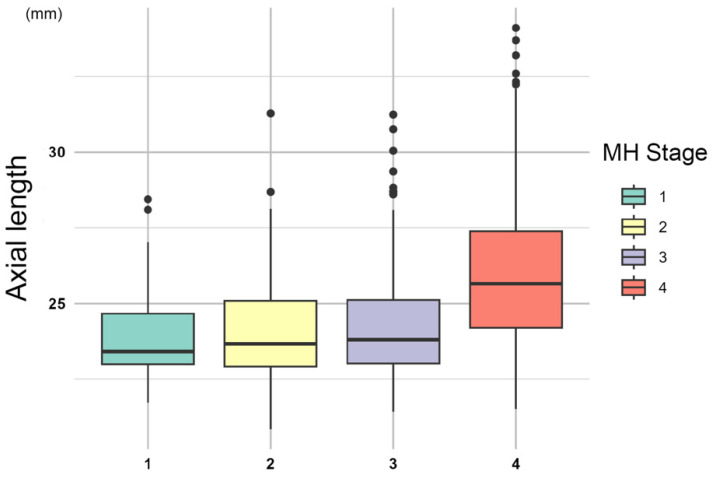
Axial length (AL) and macular hole (MH) stages. There was a tendency for the AL to increase as the MH stage progressed in both the MH2014 and MH2019 groups.

The mean AL was 24.0 ± 1.68 mm in eyes at stage 1, 24.1 ± 1.76 mm at stage 2, 24.2 ± 1.72 mm at stage 3, and 26.1 ± 2.53 mm at stage 4 (Figure 3) in both the MH2014 and MH2019 groups. There was a tendency for the AL of the eye to increase as the MH stage progressed. This was especially true for the AL of eyes at stage 4, which was significantly longer than that at the other stages (*p* < 0.001, Dunn test).

### 3.6. Ratio of Eyes with Myopic Macular Holes

The MH and ERM groups were divided into those with ALs < 23 mm, ≥23 mm but <26 mm, ≥26 mm but <30 mm, and ≥30 mm (Table 4). In the MH2014 group, 77 eyes (22.1%) had an AL < 23 mm, 196 eyes (56.2%) had an AL of 23 mm to 26 mm, 67 eyes (19.2%) had an AL of 26 mm to 30 mm, and 9 eyes (2.6%) had an AL ≥ 30 mm. In the MH2019 group, 52 eyes (16.6%) had an AL < 23 mm, 165 eyes (52.6%) had an AL between 23 mm and 26 mm, 77 eyes (24.5%) had an AL between 26 mm and 30 mm, and 20 (6.4%) had an AL ≥ 30 mm. The distribution was significantly different between the two groups (*p* = 0.014), and the number of eyes with an AL ≥ 30 mm in the MH2019 group was significantly greater than in the MH2014 group (*p* = 0.017).

On the other hand, in the ERM2014 control group, 214 eyes (21.4%) had an AL < 23 mm, 582 eyes (58.3%) had an AL between 23 mm and 26 mm, 193 eyes (19.3%) had an AL between 26 mm and 30 mm, and 9 eyes (0.9%) had an AL of ≥30 mm. In the ERM2019 group, 150 eyes (15.8%) had an AL < 23 mm, 603 eyes (63.5%) had an AL between 23 mm and 26 mm, 189 eyes (19.9%) had an AL between 26 mm and 30 mm, and 8 eyes (0.8%) had an AL ≥ 30 mm. The distribution was significantly different between the ERM2014 and ERM2019 groups (*p* = 0.014), and the number of eyes with an AL between 23 mm and 26 mm in the ERM2019 group was significantly greater than those in the ERM2014 group (*p* = 0.022).

**Figure 4:**
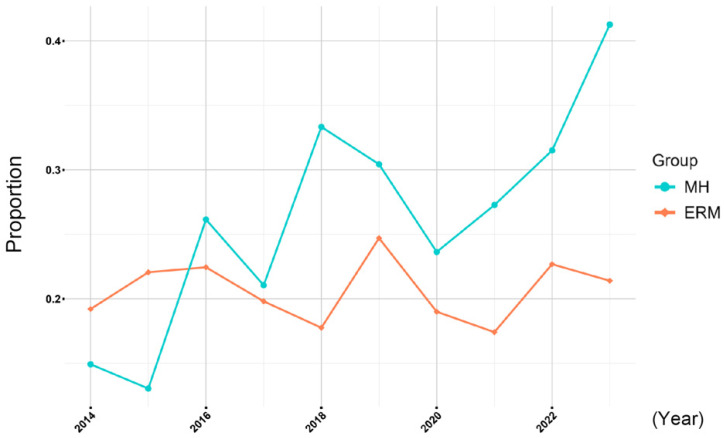
Yearly trend in myopic proportion (axial length ≥ 26 mm). The annual increase in myopia in the macular hole (MH) group is significantly higher than that in the epiretinal membrane (ERM) group.

The percentage of patients with an AL ≥ 26 mm in the MH and ERM groups by year was analyzed using a logistic regression model, and the findings are plotted in Figure 4. The annual regression coefficient for the MH group was 0.1193 (odds ratio: 1.127, 95% confidence interval: [1.060, 1.199], Table 5). The annual regression coefficient for the ERM group was 0.0020 (odds ratio: 1.002, 95% confidence interval: [0.966, 1.040]). The annual increase in myopia in the MH group was significantly greater than that in the ERM group (odds ratio: 0.889, 95% confidence interval: [0.828, 0.955], *p* = 0.0013, interaction; Year × Group). In addition, the odds of myopia increased by 12.7% per year in the MH group, while the increase was only 0.2% per year in the ERM group.

## 4. Discussion

It has been reported that the improvement in visual acuity after MH surgery was significant 6 months after the surgery, and that the mean visual acuity improved further after long-term observations of 9 months or longer [33]. In our patients, the visual acuity at the last visit was better in the MH2014 group, but there was no difference at 3 and 6 months postoperatively. These findings suggest that the difference in the duration of the observation period may have affected the results.

The results showed an increasing trend in the length of the AL in both the MH and ERM groups. The percentage of patients with an AL of ≥26 mm in the MH group also increased significantly yearly. Although the AL in the ERM group also increased significantly, its rate of increase was slower than that in the MH group. These findings suggest that the elongation of the AL was a risk factor for causing the development of an MH. In particular, the percentage of mild to moderate myopia [26] with an AL of 23 mm to 26 mm was the highest in both the MH and ERM groups. This finding is different from previous reports showing that the highest percentage was detected in normal and mildly hyperopic eyes with MHs [25,26,27,28]. In the Hisayama study, the median AL was 23.4 mm in 2005, 23.6 mm in 2012, and 23.8 mm in 2017. Thus, there was an increase in the median AL of 0.4 mm over 12 years. The AL in the ERM group was 0.1 mm over a five-year period in our patients, which may be equivalent to the AL elongation in the Hisayama study [28].

Because the period of the COVID-19 pandemic, beginning in November 2019, was included in this study, many of the institutions were forced to restrict ophthalmic surgeries until the World Health Organization declared an end to the emergency in May 2023. However, our institution continued with vitrectomy cases, and the number of surgeries during this period did not decrease significantly [34].

The incidence of posterior vitreous detachment was reported to be higher in myopic eyes with an AL of ≥26 mm than those with an AL of ≤26 mm in an age- and sex-matched case–control study [35]. In our participants, the AL was significantly longer in eyes whose MH was at stage 4 than at the other stages. The posterior vitreous detachment was more likely to progress in eyes with high myopia with an AL of ≥26 mm. This suggested that the MH formed because of the increase in the number of myopic eyes with a posterior vitreous detachment. Suda and associates [36] reported that the final MH closure rate was 100% (37/37 eyes) in patients with an AL of <26 mm after conventional ILM peeling of >3 to 4 disc diameter. However, the MH closure rate was 91.7% (11/12 eyes) in patients with an AL between 26 mm and 30 mm, and it was 0% (0/3 eyes) in patients with an AL of ≥30 mm. These findings indicated that closure of an MH was difficult to achieve in highly myopic eyes. Rizzo and associates [29] compared the standard ILM peeling and the inverted ILM flap technique for MH surgery. In their subgroup analyses of patients with high myopia with an AL of ≥26 mm, the MH closure rate was 38.9% in patients after ILM peeling, whereas the closure rate was 88.4% after the inverted ILM flap technique. They concluded that the MH closure rate was significantly higher in eyes with the inverted ILM flap technique. They suggested that the reason for this was because the inverted ILM flap reinforced the fragile retinal tissues due to elongation of the AL. In addition, the glial cells may proliferate with scaffolds to the inverted ILM tissue to reconstruct the macular microstructures. In our study, the initial MH closure rates were 93.4% in the MH2014 group and 99.0% in the MH 2019 group in patients with an AL of ≥26 mm. The closure rate was 77.7% in the MH2014 and 95.0% in the MH 2019 groups in patients with an AL of ≥30 mm. The inverted ILM flap technique has been shown to be useful for MH closure, especially in highly myopic eyes.

This study has limitations, including its single-centre, retrospective design and the restriction to the Japanese population. There was a lack of randomization of the subjects and a lack of elimination of bias in the demographic factors. This study included cases that underwent vitrectomy during the study period, so each group contained cases involving both eyes. From a clinical perspective, the onset of macular holes and their surgical management are generally considered independent events for each eye. Even when occurring bilaterally, the timing of onset, stage of macular hole, and surgical indications/techniques are often entirely different between eyes. Therefore, we consider statistical interference between eyes to be minimal. However, the complex relationship between ocular parameters in both eyes of the same patients should be thoroughly examined and addressed through rigorous scientific methods, utilizing effects modelling with the generalized estimating equations approach to analyze the dynamics of a single eye, both eyes, or a combination thereof. Because surgical methods have improved, the referral criteria may have changed, which has led to the referrals of higher-myopic eyes. The increased availability of OCT among local clinics may have led to more referrals for myopic macular holes, which are difficult to detect with conventional ophthalmoscopic examinations. The number of MH eyes with AL > 30 mm was too small to obtain meaningful conclusions. Additional studies are needed to address these limitations.

## 5. Conclusions

In conclusion, the increase in the mean AL and the rising proportion of eyes with myopic MHs indicate that the patient profile of eyes with MHs has changed over the past decade. However, the initial MH closure rate did not change significantly. We conclude that improvements to the surgical instruments and techniques are the reasons for the maintenance of the high success rates. In addition, the use of the inverted ILM flap has contributed greatly to maintaining the high closure rates, especially in highly myopic eyes.

## Figures and Tables

**Table 1:** Baseline characteristics of participants.

Category	MH2014	MH2019	*p*-Value	ERM2014	ERM2019	*p*-Value
	(2014–2018)	(2019–2023)		(2014–2018)	(2019–2023)	
Eyes (patients)	349 (333)	314 (307)	-	998 (943)	950 (899)	-
Age (year)	65.7 ± 8.66	65.2 ± 9.61	0.575 *	67.8 ± 9.42	67.5 ± 10.5	0.939 *
Laterality (right: left, eyes)	175/174	160/154	0.896 ^†^	494/504	456/494	0.538 ^†^
Sex (man/woman) (patients)	128/205	122/185	0.798 ^†^	430/513	381/518	0.179 ^†^
Phakic [eyes (%)]	315 (90%)	264 (84%)	0.023 ^†^	897 (90%)	825 (87%)	0.043 ^†^
Observation period (months)	22.0 ± 23.6	9.5 ± 9.7	<0.001 *	N/A	N/A	N/A

N/A = not applicable, *: Mann–Whitney *U* test, ^†^: chi-squared test.

**Table 2:** Preoperative and postoperative factors of eyes with a macular hole.

Category	MH2014	MH2019	*p*-Value
	(2014–2018)	(2019–2023)	
Minimum diameter (μm)	320 ± 175	297 ± 156	0.125 *
Adjusted minimum diameter (μm)	328 ± 186	309 ± 159	0.295 *
MH size (CLOSE study classification)			
Small (≤250 μm)	137	133	
Medium (>250 to ≤400 μm)	114	195	0.638 **
Large (>400 to ≤550 μm)	57	49	
X-large (>550 to ≤800 μm)	39	25	
XX-large (>800 to ≤1000 μm)	1	2	
Giant (>1000 μm)	1	0	
Base diameter (μm)	710 ± 336	687 ± 333	0.458 *
Adjusted base diameter (μm)	731 ± 357	717 ± 346	0.835 *
MH stage [eyes (%)]	stage 1: 21 (6%)	stage 1: 19 (6%)	0.001 ^†^
	stage 2: 53 (15%)	stage 2: 68 (22%)	
	stage 3: 158 (45%)	stage 3: 96 (31%)	
	stage 4: 117 (34%)	stage 4: 131 (42%)	
Baseline BCVA (logMAR)	0.45 ± 0.29	0.46 ± 0.29	0.517 *
Inverted ILM flap technique [eyes (%)]	84 (24%)	181 (58%)	<0.001 ^†^
Primary MH Closure Rate	97.7%	99.0%	0.230 **
Final MH Closure Rate	98.6%	100%	0.063 **
Postop 3 months BCVA (logMAR)	0.15 ± 0.23	0.16 ± 0.21	0.366 *
Postop 6 months BCVA (logMAR)	0.12 ± 0.22 (*n* = 272)	0.13 ± 0.21 (*n* = 180)	0.430 *
Final BCVA (logMAR)	0.10 ± 0.22 (*n* = 349)	0.13 ± 0.22 (*n* = 314)	0.008 *

MH = macular hole, ILM = internal limiting membrane, *: Mann–Whitney *U* test, ^†^: chi-squared test. **: Fisher’s exact test.

**Table 3:** Variations in axial length of eyes with macular hole and epiretinal membrane.

Axial Length	MH2014	MH2019	*p*-value
Mean ± SD (mm)	24.6 ± 2.1	25.2 ± 2.4	0.004 *
≥26 mm [eyes (%)]	76 (21.8%)	97 (30.9%)	0.008 ^†^
≥30 mm [eyes (%)]	9 (2.6%)	20 (6.4%)	0.017 ^†^
Axial Length	ERM2014	ERM2019	*p*-value
Mean ± SD (mm)	24.5 ± 1.8	24.6 ± 1.7	0.028 *
≥26 mm [eyes (%)]	202 (20.2%)	197 (20.7%)	0.786 ^†^
≥30 mm [eyes (%)]	9 (0.9%)	8 (0.8%)	0.887 ^†^

SD: standard deviation, *: Mann–Whitney *U* test, ^†^: chi-squared test.

**Table 4:** Distribution of eyes with macular hole and epiretinal membrane by axial length.

Axial Length	<23 mm	≥23 mm <26 mm	≥26 mm <30 mm	≥30 mm	*p*-Value ^†^
MH2014	22.1% (77 eyes)	56.2% (196 eyes)	19.2% (67 eyes)	2.6% (9 eyes)	0.014
MH2019	16.6% (52 eyes)	52.6% (165 eyes)	24.5% (77 eyes)	6.4% (20 eyes)	
*p*-value ^†^	0.091	0.393	0.117	0.017	
ERM2014	21.4% (214 eyes)	58.3% (582 eyes)	19.3% (193 eyes)	0.9% (9 eyes)	0.014
ERM2019	15.8% (150 eyes)	63.5% (603 eyes)	19.9% (189 eyes)	0.8% (8 eyes)	
*p*-value ^†^	0.002	0.022	0.801	0.887	

^†^: Chi-square test.

**Table 5:** Logistic regression results for myopic proportion (axial length ≥ 26 mm).

Group	Year Coefficient	Odds Ratio	(95% CI)	*p*-Value
MH	0.1193	1.127	[1.060, 1.199]	<0.001
ERM	0.0020	1.002	[0.966, 1.040]	0.0013
Interaction (Year × Group)	−0.1173	0.889	[0.828, 0.955]	0.0013

## Data Availability

The data presented in this study are available on request from the corresponding author (MI).
